# Genome Sequence of *Phlebopus portentosus* Strain PP33, a Cultivated Bolete

**DOI:** 10.1128/genomeA.00326-15

**Published:** 2015-04-23

**Authors:** Yang Cao, Ying Zhang, Zefen Yu, Pengfei Wang, Xiaozhao Tang, Xiaoxia He, Fei Mi, Chunli Liu, Dan Yang, Jianping Xu

**Affiliations:** a Laboratory for Conservation and Utilization of Bio-Resources and Key Laboratory for Microbial Resources of the Ministry of Education, Yunnan University, Kunming, Yunnan, China; b Yunnan Institute for Tropical Crop Research, Jinghong, Yunnan, China; c Department of Biology, McMaster University, Hamilton, Ontario, Canada

## Abstract

*Phlebopus portentosus* can form fruiting bodies, both independently as a saprophyte and in association with plants as an ectomycorrhizal symbiont. It thus offers an excellent model from which to examine the genetic basis of lifestyle adaptations and transitions for mushrooms. This paper reports the genome sequence of a homokaryotic strain of *P. portentosus*, PP33.

## GENOME ANNOUNCEMENT

*Phlebopus portentosus* belongs to the basidiomycete family *Boletinellaceae* and is distributed in tropical regions in Asia (1–5). Unlike its close relatives in *Boletaceae*, such as *Boletus edulis*, *P. portentosus* can be artificially cultivated (1). Indeed, this species can form mushroom-fruiting bodies, both independently as a saprophyte on artificial substrates and in association with plants as an ectomycorrhizal symbiont (1, 2, 6). This versatile fruiting strategy makes this species an excellent model organism from which to examine the genetic basis of lifestyle adaptations and the potential influences of plant factors on mushroom fruiting. The availability of a whole-genome sequence would lay a solid foundation for investigating these issues.

The genomic DNA from the homokaryotic strain PP33 of *P. portentosus* was extracted using the cetyltrimethylammonium bromide (CTAB) protocol, and three insert libraries (180-bp, 500-bp and 5-kb) were constructed. Genome sequencing was performed by the Illumina HiSeq 2000 system using 100-bp paired-end reads (Novogene Bioinformatics Technology Co., Ltd., Beijing, China) and generated 11.46 Gb of raw data. After filtering out low-quality reads, clean data of 10.90 Gb were assembled using SOAP*denovo*2 (7), with a *k*-mer size of 63. The genome was estimated to be 33.3 Mb, with a G+C content of 48%. The assembly comprised 108 scaffolds with a total length of 30.35 Mb, an *N*_50_ size of 1,450,735 bp, and an *N*_90_ size of 182,560 bp. RepeatMasker (8) was used to mask repetitive sequences, which accounted for 3.26% of the genome. The genome contained 9 rRNAs, 111 tRNAs, and 13 small nuclear RNAs (snRNAs), as revealed by tRNAscan (9) and comparisons with Rfam (10). A total of 8,390 putative protein-coding genes were predicted by Augustus (11), GeneID (12), GeneWise (13), and EVidenceModeler (14). The putative functions of the genes were derived by comparing them against several databases, including NCBI nonredundant, Swiss-Prot, TrEMBL, KEGG, and KOG. Our comparisons showed that 90.17% of the predicted genes had putative functional homologs in these databases.

The carbohydrate metabolism enzymes (CAZyme) were annotated using dbCAN (15). In total, 317 CAZymes were found, including 41 auxiliary activities (AA), 54 carbohydrate-binding modules (CBM), 48 carbohydrate esterases (CE), 107 glycoside hydrolases (GH), 60 glycosyltransferases (GT), and 7 polysaccharide lyases (PL). Interestingly, several key CAZyme families related to the degradation of lignocellulose from plant cell walls (e.g., cellulose, xylan, and pectin) were not found at all or existed only in low copies in the genome of *P. portentosus*. Some of these CAZyme proteins (e.g., GH7, GH115, CBM1, etc.) play essential roles in saprophytic fungi but are often absent in ectomycorrhizal fungi (16).

Analyses of the transcriptomes of *P. portentosus* at different developmental stages and their detailed comparisons with other saprophytic, ectomycorrhizal, and pathogenic basidiomycetes would help define and understand the genetic basis for its versatile biotrophic mechanisms.

### Nucleotide sequence accession numbers. 

This whole-genome shotgun project has been deposited at DDBJ/EMBL/GenBank under the accession no. JROP00000000. The version described in this paper is version JROP02000000.
